# Postinfectious Bronchiolitis Obliterans in Children: Diagnostic Workup and Therapeutic Options: A Workshop Report

**DOI:** 10.1155/2020/5852827

**Published:** 2020-01-30

**Authors:** Silvija-Pera Jerkic, Folke Brinkmann, Alistair Calder, Alicia Casey, Megan Dishop, Matthias Griese, Geoffrey Kurland, Mandy Niemitz, Sylvia Nyilas, Dirk Schramm, Ralf Schubert, Michael Tamm, Stefan Zielen, Martin Rosewich

**Affiliations:** ^1^Division for Allergy, Pneumology and Cystic Fibrosis, Department for Children and Adolescence, Goethe-University, Frankfurt/Main, Germany; ^2^Department of Paediatric Pneumology, Children's Hospital, Ruhr University of Bochum, Bochum, Germany; ^3^Radiology Department, Great Ormond Street Hospital for Children NHS Foundation Trust, London, UK; ^4^Boston Children's Hospital, Harvard Medical School, Boston, USA; ^5^Division of Pathology and Laboratory Medicine, Phoenix Children's Hospital, Phoenix, USA; ^6^Department of Pediatric Pneumology, Dr. von Hauner Children's Hospital, LMU Munich, German Center for Lung Research (DZL), Munich, Germany; ^7^Division of Pediatric Pulmonary Medicine, Allergy and Immunology, Department of Pediatrics, University of Pittsburgh School of Medicine, Pittsburgh, USA; ^8^University of Ulm Medical Centre, Clinic for Child and Adolescent Psychiatry/Psychotherapy, Ulm, Germany; ^9^Division of Respiratory Medicine, Department of Pediatrics, University Children's Hospital of Bern, University of Bern, Bern, Switzerland; ^10^Department of General Pediatrics, University Children's Hospital, Düsseldorf, Germany; ^11^Department of Pulmonary Medicine, University Hospital Basel, Basel, Switzerland

## Abstract

Bronchiolitis obliterans (BO) is a rare, chronic form of obstructive lung disease, often initiated with injury of the bronchiolar epithelium followed by an inflammatory response and progressive fibrosis of small airways resulting in nonuniform luminal obliteration or narrowing. The term BO comprises a group of diseases with different underlying etiologies, courses, and characteristics. Among the better recognized inciting stimuli leading to BO are airway pathogens such as adenovirus and mycoplasma, which, in a small percentage of infected children, will result in progressive fixed airflow obstruction, an entity referred to as postinfectious bronchiolitis obliterans (PIBO). The present knowledge on BO in general is reasonably well developed, in part because of the relatively high incidence in patients who have undergone lung transplantation or bone marrow transplant recipients who have had graft-versus-host disease in the posttransplant period. The cellular and molecular pathways involved in PIBO, while assumed to be similar, have not been adequately elucidated. Since 2016, an international consortium of experts with an interest in PIBO assembles on a regular basis in Geisenheim, Germany, to discuss key areas in PIBO which include diagnostic workup, treatment strategies, and research fields.

## 1. Introduction

Bronchiolitis obliterans (BO) is a rare, chronic form of obstructive lung disease. The first report of BO in 1901 by Lange described two cases of unknown origin [1]. Most early descriptions of BO were confined to case reports with autopsy findings; it was not until open lung biopsy became a more common procedure possible to describe earlier pathologic findings that suggested the cellular mechanisms that ultimately led to BO. A review of the subject of BO in children summarized available studies suggesting that BO begins with an injury of the bronchiolar epithelium followed by an inflammatory reaction which progresses towards airway fibrosis and potential luminal obliteration [2].

The term “bronchiolitis obliterans” likely describes a common pathologic alteration of small airways following a variety of inciting diseases with different aetiologies and characteristics. The initial insult, the localized inflammatory response, and preexisting factors including nutritional status and genetic variants are felt to influence the process which finally leads to the observed pathology in small airways. BO following human stem cell transplantation (HSCT), HSCT-BOS, complicated by graft-versus-host disease (GVHD) and BO following lung transplantation (LT), LT-BOS, have both been extensively studied and are relatively well understood. Postinfectious BO (PIBO) in part because of its sporadic appearance and low incidence has been much more difficult to study. While there are study-based protocols for the evaluation and potential treatment of HSCT/GVHD and LT-related BO, the diagnosis and management of PIBO are not as clear. A better understanding of the molecular and cellular mechanisms involved in the development of PIBO is needed if more directed prevention and treatment strategies are to be uncovered.

Since 2016, an international consortium of experts consisting of pediatric pulmonologists, radiologists, pathologists, physical therapists, psychologists, basic scientists, and statisticians has gathered regularly for a workshop on PIBO in Geisenheim, Germany. The workshop has been complemented by affected families and representatives of the Foundation “Stiftung Starke Lunge” (http://www.starkelunge.de). The purpose of the workshop has been to bring together international clinicians and researchers and to exchange and discuss new findings and current research data in the field of PIBO. The multidisciplinary workshop presentations have included definitions, epidemiology, etiology, and clinical courses of PIBO. The focus of the attendees has been set on specific key areas which included diagnostic workup, treatment strategies, and research fields. In conclusion, future perspectives and joint research goals were being discussed and distributed.

The following article serves as the first official workshop summary of an international consortium of experts in PIBO.

## 2. Definition

In the past, there have been several attempts to classify the condition PIBO. However, none of the present definitions are widely accepted. PIBO is a process characterised by persistent airway obstruction with functional and radiological evidence of small airway involvement that is in general unresponsive to bronchodilator treatment.

## 3. Epidemiology

PIBO is a rare disease but as there is no systematic case registration and as there are no national or international databases on PIBO, its incidence is unpredictable. The epidemiology is further hampered by a variable nomenclature. Therefore, the frequency is largely unknown and possibly more frequent than expected, as many milder cases might remain undiagnosed. It is well known that PIBO is more frequent among certain populations such as Argentinians, Native Americans, and native Koreans [3–5] pointing towards genetic factors playing a pivotal role in the initiation or perpetuation of the process.

## 4. Etiology

BO is thought to be caused by an initial insult to the lower airways [6]. However, there are three separate clinical entities of BO. The severe injury to the lower respiratory tract can be caused by either a pathogen such as adenovirus, influenza, measles, respiratory syncytial virus, and mycoplasma pneumonia (PIBO) or lung, heart (LT-BO), or bone marrow transplantation (HSCT-BO) [3, 7]. In addition, some cases of BO were triggered by toxic gases [8], chronic aspiration [9], connective tissue diseases [10], and certain drugs [11]. The repair process of restoring the epithelium and microvasculature to its previous state is severely altered.

This failure of resolution of the initial and ongoing inflammation might be an important part of the disease process in PIBO. The influx of immune and inflammatory cells and the proliferation of granulation tissue in the airways lead to bronchial obstruction and to epithelial changes such as atrophy or hyperplasia [12].

The etiology is used for clinical classification and is important to guide the investigator to the diagnosis when tachypnoea, wheezing, and hypoxaemia persist for at least 2 months after a causative event. Accordingly, most of the BO cases can be classified into postinfectious and posttransplant BO [2].

## 5. Clinical Course

The diagnosis of PIBO is usually late as the initial presentation shows a large overlap with much more frequent airway diseases, and many causative agents have been described. By the time of diagnosis, the airway disease is frequently advanced and irreversible fibrotic changes and airway obliteration have been established making the treatment difficult and often unsuccessful. As disease progression or disease stages are not defined yet, the recommendation of an appropriate treatment is further hindered by changes of inflammatory patterns over time.

**Figure 1:**
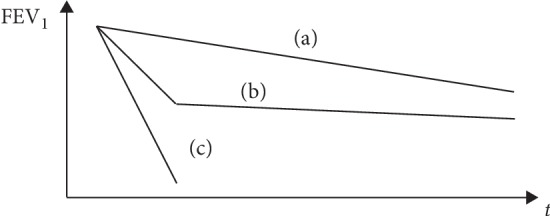
Patterns of progression in BO.

While the clinical course of BOS after HSCT has been described in the literature [13, 14], there are no existing reviews summarizing the clinical course of PIBO. The prognosis of PIBO seems to have a better outcome than HSCT-BOS and LT-BOS, respectively. However, the course varies in each case and therefore cannot be predicted. Empirical data reveals three different patterns of progression after an initial insult which were (a) an imperceptible start and slow but steady deterioration, (b) an initially rapid deterioration followed by a stable state, and (c) a rapid deterioration (Figure 1).

## 6. Diagnostic Workup

The diagnosis of PIBO is usually made by a combination of medical history, clinical findings, lung function testing, and imaging, although biopsy and histopathology remain the diagnostic gold standard.

### 6.1. Clinical Findings

PIBO is diagnosed by clinical criteria describing symptoms such as tachypnoea, cough, wheezing, exercise intolerance, and hypoxaemia persisting for at least 6 weeks after severe bronchiolitis or pneumonia with respiratory insufficiency [2]. The diagnosis is often associated with chronic obstructive lung disease after a severe viral illness in childhood [2, 3].

In case of a suggestive medical history of severe respiratory infection in a previously healthy patient, the physical examination is not very helpful in diagnosing PIBO. There are nonspecific signs which point towards PIBO such as crackles, wheezing on chest auscultation, and hyperinflation.

### 6.2. Lung Function Testing

#### 6.2.1. Spirometry

Classical spirometry is a commonly used technique which detects large airway obstruction. However, it is insensitive in small airway disease and in gradual disease progression. Pulmonary function tests usually document an obstructive impairment; however, in early stages, these tests may be normal.

**Figure 2:**
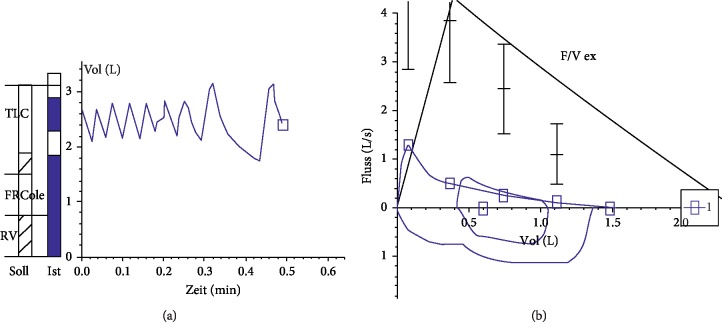
Lung function test in PIBO.

Lung function testing in patients with PIBO presents with typical patterns. The spirometry shows an irreversible or fixed obstructive flow-volume curve with decreased forced expiratory volume (FEV_1_), a reduced Tiffeneau index (FEV_1_/VC), and end-expiratory flow (MEF25). On body plethysmography, hyperinflation and air trapping are indicated by an increased residual volume (RV) and an increased functional residual capacity (RV/TLC) [5, 15]. Typically, there is either no or little response to bronchodilatation [16]. Patients with PIBO tend to have milder diseases than those with BOS following bone marrow transplantation [3]. (Figure 2) A cohort study by Colom et al. reported severely impaired pulmonary function among children with PIBO after long-term follow-up [17]. Disproportional growth of lung parenchyma in airways of affected children was a proposed explanation.

#### 6.2.2. Multiple Breath Washout

Spirometry primarily measures obstruction in the larger airways; however, it is a generally insensitive detector of small airway obstruction. A joint task force formed by the International Society for Heart and Lung Transplantation (ISHLT), the American Thoracic Society (ATS), and the European Respiratory Society (ERS) has published a practice guideline of diagnosis and management of BOS post-LT. It defines BOS as a small airway disease diagnosed with a persistent decrease in FEV_1_ of at least 10–20% compared to the mean of the two best postoperative values after lung transplantation in the absence of any other identifiable causes [18]. Because biopsy is invasive and associated with risk of bleeding and further complications, the National Institute of Health (NIH) Consensus proposed new criteria for diagnosis and scoring the severity of chronic GVHD of the lungs pointing towards BOS [19]. The clinical diagnosis of BOS post-HSCT is made when FEV_1_/VC < 0.7, FEV_1_ < 75% of pred. with >10% decline over less than 2 years, absence of infection, and evidence of air trapping by CT or lung function testing [19]. However, diagnosing and scoring the severity of BOS remains challenging due to limited understanding of pathophysiology, and current guidelines for BOS point out the need to find new sensitive lung function indices to detect early small airway impairment [18].

In the last decade, the usability of new inert gas washout measurements in different lung diseases gained worldwide attention. Nitrogen multiple breath washout (N2-MBW) is a feasible and sensitive tool to detect early small airway impairment in chronic lung diseases in children and adults [20–24]. The main outcomes detect global (lung clearance index: LCI) as well as acinar (Sacin) ventilation inhomogeneity. Previous studies showed that the lung clearance index (LCI) is already abnormal in the majority of patients with cystic fibrosis (CF), primary ciliary dyskinesia (PCD), and chronic obstructive pulmonary disease (COPD) even though spirometry indices are still in the normal range [20, 24–27]. The N2-MBW derived Sacin correlates closely with impaired ventilation in the distal lung compartment, the acinus [28], and is highly elevated in adults and children with small airway disease [29–31].

Lung clearance index (LCI) measured by multiple breath washout (MBW) is likely to be of benefit in PIBO. The spirometry aids to diagnose PIBO in children, and it detects irreversible bronchiolar obstruction. However, spirometry in very young children with PIBO is not feasible due to limited cooperation. A recent prospective study by Kim et al. evaluated the utility of LCI assessing the degree of small airway obstruction in children with PIBO [32]. Twenty infants diagnosed with PIBO underwent pulmonary lung function tests, MBW, and chest CT. The concordance indices of LCI were significantly correlated with the air-trapping lung volume percentage from CT which suggests that LCI is a feasible, complimentary tool for assessing children with PIBO.

A study in thirty-seven pediatric patients >100 days after lung transplantation showed highly elevated LCI values in comparison with FEV_1_/FVC and a poor agreement between both parameters [33, 34]. In line with this, another study which enrolled 15 patients, 5 of whom already had a diagnosis of chronic lung allograft dysfunction (CLAD), showed that the LCI identified lung allograft dysfunction in more patients than the use of standardized spirometric measures [35].

These results indicate as well that a combination of different lung function parameters may be more predictive of disease course and prognosis than any single parameter and that LCI should be established as a useful complementary parameter in assessing patients with PIBO. Further longitudinal studies will show if inert gas washout indices can be used as an early predictive outcome to detect early PIBO. At present, LCI, which is the main outcome parameter from the N2-MBW, detects global ventilation inhomogeneity with good sensitivity. In order to analyse the specificity, it is recommendable to measure N2-MBW during clinical routine accompanied by commonly used lung function test on a regular basis on one to three monthly follow-ups. Hence, N2-MBW indices could be used as parameters to predict patient's outcome. Furthermore, N2-MBW could be used for clinical research to assess the treatment effects of therapeutic interventions in PIBO.

#### 6.2.3. Forced Oscillation Technique

The forced oscillation technique (FOT) is a noninvasive method with which to measure respiratory mechanics [36]. The ERS formed a joint ERS task force which led to publication of a practice guideline on clinical application of FOT in 2003 [36].

Especially for the pediatric population, FOT has the advantage over spirometry that it does not require the performance of respiratory manoeuvres due to the small-amplitude pressure oscillations superimposed on the normal breathing. Moreover, in contrast with spirometry where a deep inspiration is needed, forced oscillation technique requires minimal cooperation and does not modify the airway smooth muscle tone. For preschool children and children unable to successfully perform spirometry, it can become difficult to understand and perform forced expiratory manoeuvres, and therefore, spirometry results may not be evaluable. A study by Evans et al. showed that respiratory impedance outcomes were significantly impaired in three groups of children with asthma, CF, and children born preterm, respectively, compared to healthy controls [37]. However, their findings also showed that the utility of specific FOT outcomes is dependent on the respiratory disease being assessed, and therefore, a more detailed disease-specific approach to interpreting FOT would be essential. There are no studies looking at the feasibility of FOT in PIBO: however, it would be an interesting tool to look at in preschool children patients with PIBO. Further studies are needed to demonstrate whether FOT is a useful complementary technique in identifying and assessing young pediatric patients with PIBO.

## 7. Imaging

### 7.1. Pathobiological Basis of CT Findings in PIBO

**Figure 3:**
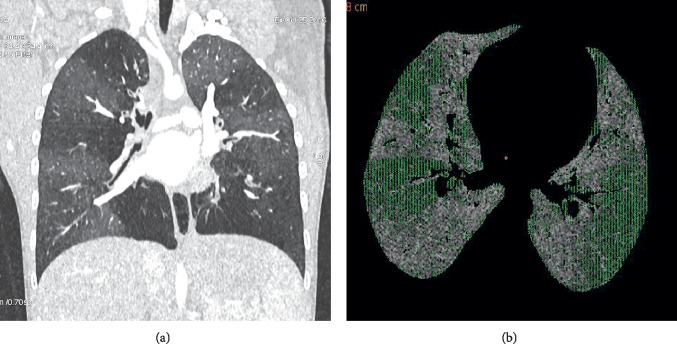
CT chest in PIBO.

The principal finding in PIBO is variation in the density of alveolar lung tissue, termed “mosaic attenuation.” Diseased lung shows lower density through two principal mechanisms, alveolar hyperinflation and hypoxic vasoconstriction [38]. Histological studies suggest that hyperinflation can occur with both complete and incomplete bronchiolar obstruction; hyperinflation in the presence of complete bronchiolar obstruction must reflect a “check-valve” mechanism of collateral air circulation [39]. Hypoxic vasoconstriction leads to redistribution of blood flow to “healthy” lung. When bronchiolar disease is extensive, a larger volume of blood will be redistributed to a smaller volume of healthy lung, which will become denser as a result, demonstrating ground-glass attenuation. Thus, in extensive disease, mosaic attenuation is accentuated (Figures 3(a) and 3(b)).

### 7.2. CT Techniques for Evaluating PIBO

There are a number of decisions to be made in selecting a CT technique in this setting.

Adult studies suggest that performing inspiratory CT only will miss some cases of bronchiolitis obliterans and that adding in expiratory sections increases sensitivity [40, 41]. Interestingly, those with abnormalities on expiratory scans had milder disease. Obtaining expiratory sections reliably in young children (under age 6) generally requires general anaesthesia. An alternative for “free-breathing” scans is to perform lateral decubitus acquisitions, with the dependent lung acting as the “expiratory” lung. Although there is evidence that this may be effective [42], workshop attendees' experience is that this is not always so in clinical practice.

Postprocessing techniques during image interpretation can enhance the detection of mosaic attenuation. The use of minimal intensity projection (minIP) is one such method which may be effective [43]. A critical factor is the selection of window settings. Lung images are typically evaluated using a wide window width (1200–1500), but mosaic attenuation can be much more easily detected with a narrower window width (∼300).

Historically, CT has been difficult to perform in young children. Whilst infants could sometimes be scanned asleep after feeding (“feed and wrap”), children between the ages of 3 months and 4–6 years generally required general anaesthesia to perform thoracic CT imaging. However, the advent of ultra-high-speed scanning allows a much lower utilisation of general anaesthesia. Some modern scanners can perform scans of the entire thorax in approximately 0.3 seconds. This can almost eliminate artefacts from motion and breathing. When combined with gentle immobilisation, the use of general anaesthesia for chest CT scanning in 1- to –6-years-olds in one centre practicing these methods has fallen from 50% to 10%. A downside of avoiding general anaesthesia is the inability to select the respiratory phase, but this can be ameliorated by the techniques described above.

Another traditional aspect of CT scanning for PIBO and other forms of diffuse lung disease has been the use of interrupted as opposed to contiguous volume acquisitions. The purpose of interrupted sections (typically 1 mm sections at 10 mm intervals) is to reduce radiation dose. However, the reduction in information can lead to reduced sensitivity and precision [44], and, as other radiation dose reduction techniques are available, this technique is much less frequently performed today.

The use of intravenous contrast is also discussed. In general, this can be avoided in the initial diagnosis of PIBO, avoiding the need for cannula placement. It is unlikely that IV contrast either enhances or interferes with the diagnosis of PIBO, but its use may be important in severe or atypical disease, for example, in children with pulmonary hypertension, those being considered for lung transplantation, or those who may have lymphadenopathy.

Radiation dose from CT scanning is a major limitation on its use. Fortunately, a range of modern innovations has seen the dose from CT fall dramatically, and where historically, radiation doses were in the order of 10 mSv, and it is now possible to scan at doses of well under 1 mSv. For comparison, annual background radiation dose in the UK is approximately 2.7 mSv. The risks of such level of radiation are unknown, and a commonly used approach applying the so-called “linear no-threshold model” suggests that a 0.5 mSv exposure at age 1 would convey a lifetime excess cancer risk of 1 : 20000 [45]; this is set against a background lifetime cancer risk for children born today approaching 1 : 2. However, increasingly, it is being suggested that low radiation exposure may not carry any excess risk at all [46]. Potential alternatives to CT as an advanced imaging tool for PIBO include various forms of MRI scan (see below).

### 7.3. The Role of CT in the Modern Diagnosis of PIBO

CT plays a central role in the diagnosis of PIBO; the finding of mosaic attenuation on CT is the strongest predictor of PIBO (along with a typical clinical history) [47].

PIBO typically manifests a clear clinical history, and as such, differential diagnoses often do not need to be considered. However, if disease onset is insidious or if there are other complicating features, then other entities are sometimes considered, including cystic fibrosis, other causes of severe bronchiectasis and severe asthma.

In general, these are best diagnosed by nonradiological means, as there can be considerable overlap in the CT findings. An adult study suggested that mosaic attenuation on inspiratory CT was the best distinguishing factor of adult BO (of undefined etiology) from severe asthma [48]; alternatively, a study in children suggested that the presence of bronchial dilatation/bronchiectasis, present in 88% of children with PIBO, but only 29% with severe asthma, was a better distinguishing criterion [49]. Another possible role for CT in PIBO is as a marker of disease severity. Several studies have demonstrated correlation between various CT measures of disease severity and lung function measures in BO of mixed etiologies [50–52], but none of these suggest compelling support for CT as a severity measure. Applying automated and semiautomated approaches is likely to enhance the precision of CT measures of lung severity. Two such approaches are (1) measuring the volume of “air-trapped” lung using a “density mask,” an adjustable segmentation threshold [32, 53], and (2) parametric response mapping, whereby voxels on paired inspiratory and expiratory scans are matched and scored according to the densities on each [54].

### 7.4. MRI in PIBO

MRI imaging of the lungs has undergone significant developments in recent years and has attracted interest for pediatric imaging in particular in view of the lack of ionising radiation. Structural lung imaging with MRI is challenging owing to low signal-to-noise ratio and the poorer spatial resolution of MRI compared with CT. Newer sequences however, particularly ultra-short echo time (UTE) sequences, have been shown to produce excellent quality images in a range of lung pathologies, including airway disease such as cystic fibrosis [55]. MRI also has the added advantage of greater capacity for functional imaging. MRI ventilation imaging of the lungs using hyperpolarised gases (e.g., He^3^ Xe^129^) has been shown to be exquisitely sensitive for small airways disease in CF [56] and would appear to be a logical choice for PIBO. However, these techniques are not widely available, requiring bespoke equipment to generate the gas agents, and specialised coils and MRI systems. Alternative approaches to MRI ventilation imaging include using 100% oxygen and mapping its effect on T1 signal [57] and using multivolume MRI sequences to explore changes in MRI signal intensity over the respiratory cycle and thereby derive ventilation maps [58, 59]. There are several drawbacks to MRI: firstly, it has not been systematically evaluated in the setting of PIBO as opposed to other forms of chronic airway disease (e.g., chronic lung allograft dysfunction and CF) or in younger children. Secondly, lung imaging generally requires additional sequences, agents, or equipment not widely available and in many centres is only available on a research basis; finally, and most importantly, MRI almost always requires general anaesthesia in those under the age of 5-6 years, whilst PIBO tends to have its first onset at age 1-2 years.

### 7.5. Concluding Comments

CT has a clear, logical role in the diagnosis of PIBO. However, the scientific evidence base for this is somewhat limited and in large part derives from studies in adults with noninfectious etiologies of PIBO. There are a number of open questions in this field. Critically, imaging for PIBO tends to be performed sometime after the initial pulmonary insult (typically adenovirus). It is likely that the main window for modulating the outcome in PIBO is earlier than this. An ability to distinguish uncomplicated from complicated adenovirus pneumonia at the time of initial infection is needed if future interventions are to be correctly targeted (e.g., immunomodulation); it is unknown whether imaging might play a role in this. Outcomes in PIBO are variable, with about 20% of cases progressing to a severe or fatal outcome and 20% making a full recovery; whether imaging can predict these outcomes is currently unknown. Finally, if early intervention studies are to be carried out in the future, CT may be proposed as a surrogate outcome measure; however, further validation for such a role would need to be carried out. MRI has some advantages over CT as an imaging tool for small airways disease, but is yet to be validated and is often impractical, in the younger children with postinfectious BO.

## 8. Histopathology

The term bronchiolitis obliterans as a histopathological entity was classified with two distinct types of bronchiolar involvement. It includes the constrictive type with peribronchial fibrosis and the proliferative type with airway obstruction by intraluminal polyps of inflammatory granulation tissues [60].

Lung biopsies have been considered the gold standard for diagnosing BO. Typically, these biopsies show a progressive inflammatory response with elements of tissue remodelling, fibrosis of the small airways, and airway obstruction. However, due to the heterogeneous distribution of airway involvement throughout the lung parenchyma and the variability of chronic inflammation among patients, these biopsies can lead to sampling error which reduces the sensitivity. In addition, there are only some similarities at the initial airway injury and early healing phase such as lymphocytic inflammation in patients with PIBO, LT-BO, and HSCT-BO. The end-stage pathology reveals the same fibrotic airways with airway dropout.

In this regard, BO remains a joint diagnosis considering clinical, radiological, and histopathological features.

**Figure 4:**
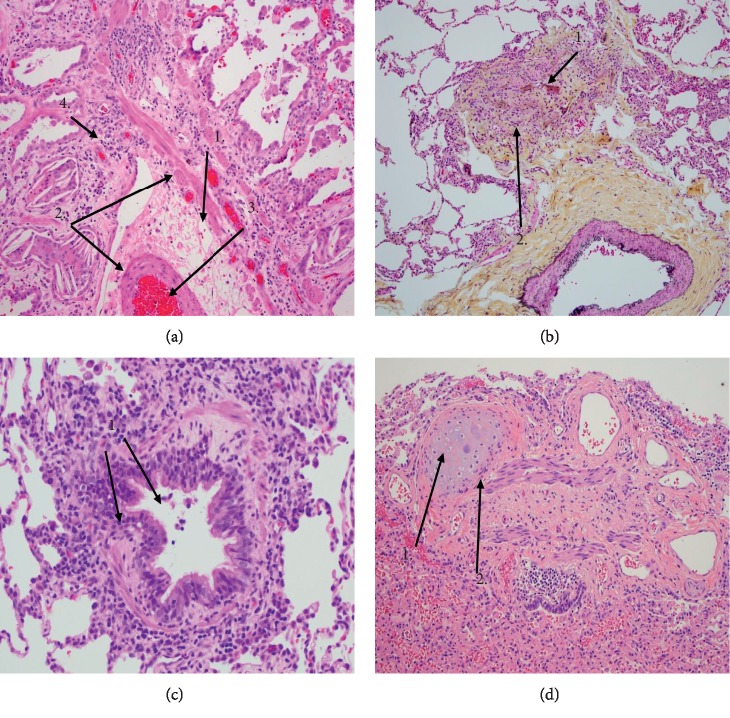
(a) Haematoxylin and eosin staining (10x original magnification). (1) Cholesterol clefts; (2) smooth muscles; (3) luminal obliteration; (4) scattered lymphocytes. (b) Movat pentachrome (10x original magnification). (1) Unpaired artery branch; (2) obliterated bronchiole. (c) Haematoxylin and eosin staining (20x original magnification). (1) Constrictive bronchiolitis with subepithelial fibrosis and cellular infiltrates. (d) Haematoxylin and eosin staining (10x original magnification). (1) Cartilage island; (2) fibromuscular scar.

Typical lesions are presented in the following figure (Figure 4).

Figure 4(a) shows typical lesions of obliterative bronchiolitis, demonstrating a preserved smooth muscle wall with luminal obliteration by fibroconnective tissue and scattered lymphocytes. There is no residual mucosa. Peribronchiolar accumulation of macrophages and cholesterol clefts are common secondary findings resulting from small chronic airway obstruction and may be a clue to the diagnosis at low power.

Figure 4(b) shows at screening magnification the finding of an “unpaired” pulmonary artery branch which allows recognition of an adjacent obliterated bronchiole.

In Figure 4(c), constrictive bronchiolitis can be seen which indicates narrowing of a bronchiole lumen by varying degrees of subepithelial fibrosis, with or without cellular infiltrate. This example shows mild constrictive bronchiolitis as part of the spectrum of pathology in bronchiolitis obliterans syndrome.

Figure 4(d) is an example that obliteration of a bronchus is recognized by an island of cartilage adjacent to a fibromuscular scar. “Bronchiolitis obliterans” may be seen following severe necrotizing respiratory viral infection, such as influenza, and is a common end-stage manifestation of Stevens–Johnson syndrome.

## 9. Treatment Options

Since PIBO is a rare chronic irreversible obstructive lung disease, treatment options have not been clearly defined and there are different strategies between centers. Most of the current knowledge comes from studies looking at patients with BOS either post-LT or post-HSCT [61].

The treatment of PIBO is empirical, and there is no accepted treatment protocol. However, there are a few randomized placebo-controlled trials in BOS. Most of the reports published on treatment options suffer from small patient numbers, absence of controls, and diverse patient groups at the start of therapy.

In general, the treatment for PIBO should be a combination of optimal supportive care and anti-inflammatory therapy to impair lymphocyte proliferation and activation since inflammation plays an important role in the pathogenesis of PIBO [62] (Table 1). Nevertheless, it is common consensus that before systemic anti-inflammatory treatment is given, a diagnostic workup including bronchoscopy with bronchoalveolar lavage (BAL) should be performed thoroughly to rule out persistent infections with viral, fungal, and bacterial pathogens.

It is important to note that a study analysing sleep-disordered breathing in children with PIBO showed that the risk of nocturnal hypoxia was increased in patients with PIBO and it was correlated with the severity of lung disease determined by pulmonary function test. While the duration of central apnoeas was shorter, they were more prone to desaturations [63].

### 9.1. Efficacy of Steroids

In dependence on the clinical course, inhaled and systemic corticosteroids are used to counteract the inflammatory component. Ideally, corticosteroids should be given early during the developing disease process and before airway fibrosis is established [15]. It is common agreement that the approach of choice is pulse steroid therapy with intravenous methylprednisolone 10–30 mg/kg for 3 consecutive days and repeated monthly for 3–6 months as it is used for childhood interstitial lung disease. Oral corticosteroids and an elongated course of systemic corticosteroid should be avoided, since this is associated with severe side effects and complications like bone fractures or mortality from infections. Some centers report that during the vulnerable time of the corticosteroid pulses, they would give intravenous immunoglobulins as supportive treatment to prevent infections. However, there are no published data available to demonstrate the benefit of such immunoglobulin treatment.

The available data on corticosteroid efficacy in PIBO are rather limited (Table 2). Tomikawa et al. studied 40 patients who were diagnosed with PIBO and who were treated with methylprednisolone pulse therapy in monthly cycles and additional inhaled corticosteroids for the follow-up period [67]. The frequency of wheezing exacerbations after 24 months of methylprednisolone pulse therapy and frequency of hospitalization after 18 months were significantly reduced and oxygen saturations improved. Yoon et al. evaluated the CT features that predict responsiveness to methylprednisolone pulse therapy. The authors concluded that children with PIBO may respond favorably to pulse therapy when pretreatment CT indicates bronchial wall thickening [68]. Although corticosteroids are effective in acute deterioration of PIBO and in acute graft-versus-host disease (GVHD) by improving lung function and exercise capacity, the long-term benefit is largely unknown. In view of the unclear effect of corticosteroids upon the long-term clinical outcome, their substantial toxicities, and a high risk for serious and fatal infections, a noncorticosteroid treatment for long-term management is recommended.

### 9.2. Efficacy of Azithromycin

It is well known that corticosteroids do not target neutrophilic airway inflammation efficiently. In contrast, azithromycin has been effective in controlling neutrophilic inflammation and improving lung function in various diseases such as diffuse panbronchiolitis, cystic fibrosis COPD, and BOS post-lung transplantation [69–71]. A randomised controlled trial of azithromycin therapy in BOS post-lung transplantation by Corris et al. improved FEV_1_ in patients with BOS post-LT and appeared superior to placebo [72]. A pilot study of six patients by Gerhard et al. showed that azithromycin improved FEV_1_ in five patients with BOS post-LT with a mean increase of 17.1% predicted over a 4-month period [73]. Verleden revealed that azithromycin improved FEV_1_ and decreased airway neutrophilia and IL-8 levels in patients with reversible neutrophilic inflammation in post-LT-BOS [71]. The exact mechanism by which azithromycin downregulates the host inflammatory response is largely unknown. Different mechanisms have been described so far such as reduced microbiologic burden, decreased numbers of airway neutrophils, suppression of IL-8, and other neutrophil chemotactic cytokines and interference with neutrophilic function.

Apart from reports with significant clinical response to azithromycin, there are studies where azithromycin therapy did not show any improvement in lung function. A systemic review and meta-analysis on the impact of azithromycin on change in FEV_1_ shows that there was no sufficient evidence to support or refute the use of azithromycin in the treatment of patients who develop BOS post-HSCT [74].

With regard to azithromycin as treatment for PIBO, the literature is very scarce (Table 3). A study by Li et al. looked into 42 cases of children diagnosed with PIBO who revealed some benefit of being treated by a combination of systemic corticosteroids and oral azithromycin [64]. Although there are no RCTs in children with PIBO, oral azithromycin 10 mg/kg given three times weekly is recommended on the basis of studies in other obstructive diseases [15].

Although there are no RCTs in children with PIBO and since azithromycin is well tolerated, oral azithromycin 10 mg/kg given three times weekly is recommended as long-term management on the basis of studies in other obstructive diseases [15].

### 9.3. Efficacy of Fluticasone, Azithromycin, and Montelukast (FAM)

In a pilot study, montelukast has been tried in fibroproliferative post-lung transplant BOS and showed slowing of decline in FEV_1_ [87]. However, a recent trial by Ruttens et al. showed no additional survival benefit with montelukast compared with placebo [88]. Several studies reported that a combination of inhaled fluticasone, azithromycin, and montelukast (FAM) could be an effective treatment in patients with BOS [89].

Recently, a phase II, open-label, multicenter study evaluated the treatment effect of FAM in combination with a steroid pulse at the start of therapy. The primary endpoint was treatment failure, defined as 10% or greater FEV_1_% decline at 3 months [90]. At 3 months, only 6% vs. 40% historical controls (*p* < 0.001) had treatment failure. These data suggest that FAM was well tolerated and that treatment with FAM and steroid pulse may halt pulmonary decline in newly diagnosed BOS post-HSCT.

The efficacy of FAM in PIBO is not known. Single centers have reported to use FAM as a combination therapy in PIBO. Although this treatment option is safe, no formal trials have been conducted yet.

### 9.4. Efficacy of Bronchodilator Treatment

Chronic obstructive airway disease and hyperinflation play an important role in the pathophysiology of PIBO. Typically, these patients do not show a significant postbronchodilator response after inhaling 400–600 *μ*g salbutamol. In the recent study of Rosewich et al., 5 out of 20 patients showed postbronchodilator changes consistent with reversibility based on FEV_1_ (12% increments and 200 ml) [62]. Whether this is due to concomitant allergies or asthma or whether it is a symptom of a distinctive subtype of PIBO is difficult to say. Two studies reported that patients do favourably respond to long-acting muscarinic receptor antagonists (LAMAs). Interestingly the placebo-controlled trial of Teixeira demonstrated a meaningful improvement after tiotropium alone in PIBO [91] and after tiotropium plus salbuterol in BOS post-HSCT [92]. In a multicentre study with adult patients by Bergeron et al., it was revealed that there was a significant improvement in FEV_1_ in patients with mild to severe BOS post-HSCT after a 6-months trial of budesonide/formoterol [93].

### 9.5. Potential Future Treatment Options

There has been some debate about future treatment options. However, these options are still being assessed and further research studies are required. There are some recent preclinical and clinical studies of lung diseases using mesenchymal stem/stromal cells (MSCs). MSCs have shown immunomodulatory and anti-inflammatory properties and have proven some efficacy in various lung injury models in animals. However, they can also have profibrotic effects. There are multiple studies with animal models using MSCs to treat BOS [94–98]. Cao et al. showed that in seven patients with BOS and respiratory failure, six of them presented with lung function improvement 6 months after they were given i.v. MSCs for 4 weeks along with budesonide, salmeterol, and methylprednisolone [99].

Extracorporeal photopheresis (ECP) [100] and total lymphoid irradiation (TLI) [101] have been used to treat BOS post-LT with variable efficacy. ECP did show reduction of lung function decline and was well tolerated in a single center with 10 years of experience [102].

The treatment of BOS post-HSCT remains difficult. It has been reported that infliximab, a monoclonal antibody directed against tumor necrosis factor-*α* (TNF-*α*), has been successfully used in a child with BOS post-HSCT. Further treatments including inhaled cyclosporine and clotrimazole might become an option; however, additional trials and studies need to be conducted.

## 10. New Research Fields

The current knowledge of the underlying mechanisms of PIBO is scarce and treatment is challenging. Multicentre research is essential to understand the exact mechanisms of PIBO, be able to identify biomarkers and clarify monitoring and treatment. As PIBO affects mainly children, further research and clinical trials need to include pediatric patients.

The international consortium of clinical and scientific experts in PIBO has made a multidisciplinary effort to set up new, collaborative research approaches. The focus was set on the following three areas of investigation.

### 10.1. MicroRNA

New techniques such as next-generation sequencing (NGS) for sequencing large amounts of data have fundamentally changed the viewpoint on scientific and diagnostic questions. MicroRNAs (miRNAs) play an important role in the pathogenesis of numbers of respiratory diseases such as asthma, CF, idiopathic interstitial pneumonia, and COPD. miRNAs are small noncoding RNAs that control gene expression posttranscriptionally by inhibiting translation, and thus, they play a central role in the epigenetic modification of gene expression. In addition, their promising potential as biomarkers and basis for the development of new classes of therapeutic compounds has been described recently [103, 104].

In view of these findings, the assumption has been raised whether miRNAs could play an important role in the process of inflammation and development of fibrosis in PIBO. Further research is required to evaluate the role of miRNA in PIBO.

### 10.2. Predictive Biomarkers

Distinctive inflammatory profiles have been sought to improve our understanding of ongoing inflammation and remodelling in airway diseases such as cystic fibrosis and asthma [105–108].

Equivalent data for PIBO are lacking, in part because of an absence of reproducible animal models for which potential biomarkers can be determined and in part because of the rarity of PIBO following life-threatening infections. Adenoviral lower respiratory infections are a recognized risk factor for the development of PIBO, yet the incidence of this complication in children with documented adenovirus is quite variable ranging from less than 5% [109, 110] to greater than 25% in certain populations [111, 112]. The reason for this wide difference in incidence remains unclear, although variations in viral prevalence and virulence have been suggested as potential explanations [113].

The search for biomarkers that potentially could be useful in predicting PIBO following adenoviral or mycoplasma pulmonary infections has centred on studies of patients with life-threatening respiratory complications of these pathogens [62, 114, 115]. Potential biomarkers relevant to the underlying disease process in PIBO are listed in Table 3.

Based on these studies as well as investigations of animal models of BOS following lung or stem cell transplantation, there are felt to be several factors involved in the development of PIBO: (a) the virulence of the inciting pathogen itself, possibly serotype-specific, causing airway epithelial injury; (b) cytokines including IL-6, IL-8, and TNF- *α*; (c) other molecular components which are upregulated locally; and (d) cellular components, either locally “in situ” or directed by cytokines and other mechanisms to the region of airway damage.

Through an orchestrated interaction, the airway epithelial damage, rather than repairing it in a normal fashion with a normal epithelial layer, is largely replaced by T cells and neutrophils, with subsequent matrix degradation, collagen deposition, and fibroblast stimulation, ultimately leading to the airway fibrosis characteristic of BO [6].

The lack of a good animal model of PIBO hampers the efforts to determine which of the many potential biomarkers would serve as a reasonable potential target in human trials. An example of the difficulties of a possible animal model is that of mouse adenovirus-1 (MAV-1) respiratory infections in neonatal mice. The extensive and careful work of Weinberg and his colleagues has shown that neonatal mice thus infected have higher viral loads than adult mice. In addition, infected neonatal mice have impaired recruitment of inflammatory cells compared with adult mice. Yet, perhaps paradoxically, viral clearance is unimpaired in neonatal mice [116]. There are multiple interactive cytokines that are involved in viral clearance as well as inflammatory cell recruitment in this animal model [117, 118].

Further, neonatal mice surviving MAV-1 respiratory infection do not show histologic changes suggestive of bronchiolitis obliterans. Thus, an animal model, even one with reproducibility and demonstrable alterations in biomarkers, may not be useful for a study of PIBO if the final histologic changes are lacking.

It is likely that as other molecular tools, such as miRNA, next-generation sequencing, or whole transcriptome sequencing, are perfected, we will be better able to establish the correct targets for study and, potentially, directed pharmacotherapy to either predict an increased risk for PIBO in risk patients or to treat those patients already developing PIBO.

### 10.3. Microbiome

In recent years, the field of human microbiome research has been growing exponentially including the microbiome analysis of the respiratory system. It has been suggested that certain microbes play important roles in the development of healthy immune responses and that, on the other hand, microbial dysbiosis can contribute to chronic inflammatory lung diseases such as asthma, COPD, and CF [119]. Further studies presented data about disordered or decreased microbial communities in asthma, COPD, and CF [120–122]. Although microbiome data in PIBO are scarce, studies of bronchiolitis obliterans syndrome (BOS) demonstrated that both bacterial and viral infections are associated with elevated risk of BOS and that posttransplantation infections are associated with a worse outcome [123].

Usual procedures like microbiological culture, serology, point-of-care tests, or PCR can be successful but have weak points including low sensitivity and specificity, and often it is difficult to distinguish between colonization and infection. From the onset of the diagnosis, regular PIBO microbiome analysis would be needed to comprehend microbiome-driven pathophysiology and inflammation in PIBO.

## 11. Patient's Perspectives

One strength of this workshop is the engagement of the affected families through the foundation “Stiftung Starke Lunge.” The families have been asked to give an overview of their personal perspectives, especially their view on diagnostic delay, treatment effectiveness, quality of life, and the impact on daily functioning.

The patient's perspectives are shown in Table 4. The foundation “Stiftung Starke Lunge” has created a web page (http://www.starkelunge.de) dedicated to PIBO to inform and to support patients, their families, and medical professionals. This German web page will be soon available in other languages.

## 12. Future Directions

Postinfectious bronchiolitis obliterans (PIBO) in children is a rare chronic irreversible obstructive pulmonary disease that can follow any of several infections. The diagnosis is made collectively by clinical, radiological, and physiological findings and a history suggestive of PIBO. However, there are obvious differences in individual outcomes and the natural course cannot be predicted. While some children are left with serious structural and functional lung disease, others experience more subtle effects. Further multicenter prospective trials are required to understand the complexity of the disease and to define risk factors. Future effort should focus on establishing supplemental specific diagnostic markers and ultimately a tailored approach of individual treatment options.

Better understanding of the entity PIBO will lead to better counselling of the affected patients and their families and will support these children and adolescents with a future management plan for their lifelong respiratory disease.

## Figures and Tables

**Table 1:** Treatment options in PIBO.

Anti-inflammatory therapy	Supportive care
(i) Systemic corticosteroid	(i) Supplemental O2
(ii) Azithromycin	(ii) Nutritional support
(iii) Combination-therapy: FAM (fluticasone/azithromycin/montelukast)	(iii) Immunization (influenza/pneumococcal)
(iv) Immunglobulin substitution	(iv) Avoid cigarette smoke
(v) Steroid sparing anti-inflammatory agent	(v) Airway clearance if bronchiectasis (hypertonic saline)
(vi) Tumor necrosis factor inhibitor	(vi) Bronchodilators if responsive
(vii) Rescue therapy (extracorporeal photopheresis)	(vii) Exercise therapy/pulmonary rehabilitation

**Table 2:** Corticosteroids in PIBO.

Study (year) journal	Li et. al. (2014) BMC Pediatrics [64]	Chen et. al. (2012) Chinese Journal of Pediatrics [65]	Wang et. al. (2015) Experimental & Therapeutic Medicine [66]

Number Comparative design Study design	42 children No control group/no placebo Prospective cohort	26 children No control group/no placebo Retrospective cohort	16 children No control group/no placebo Retrospective cohort

Treatment regime	Oral prednisone (1.5 mg/kg/day with weaning for 6–9 mo) and azithromycin (5 mg/kg for 3 d/wk for 6 mo) Some also had i.v. steroids during acute phase	Oral steroid and azithromycin	Oral prednisolone (1 mg/kg/day with dose reduction of 1.25 mg/month) and azithromycin (7.5 mg/kg twice weekly) Variable treatment duration

Assessment of outcome	Effective (subjective/objective measures PFT <10% decline) or ineffective		Clinical symptoms and HRCT findings

Effectiveness	Effective in 86% at 6 months		10 of 16 improved

**Table 3:** Potential biomarkers in BO.

Parameter	Entity	Serum	BAL/sputum	Evidence	Reference
Neutrophils	PIBO	—	Increased	A	Eckrich et al. [75]
	BOS				
CRP	BOS	Increased	Increased	A	Vos et al. [76]
IL-1*β*	PIBO	—	Increased	A	Rosewich et al. [62]
	BOS				
IL-6	PIBO	—	Increased	A	Rosewich et al. [62]
	BOS				
IL-8	PIBO	—	Increased	A	Koh et al. [77]
YKL-40	PIBO	Increased	—	B	Jang et al. [78]
KL-6	BOS	Increased	—	B	Ohshimo et al. [79]
Surfactant protein D	BOS	Decreased	—	B	Nakane et al. [80]
Metalloproteases 8 + 9	BOS	Normal	Increased	B	Taghavi et al. [81]
IL-17/IL23	BOS	—	Increased	B	Vanaudenaerde et al. [82]
CD8 T	BOS	Increased	Increased	B	Hodge et al. [83]
NK cells	BOS	Increased	Increased	B	Hodge et al. [83]
CXCL9	BOS	—	Increased	B	Shino et al. [84]
miRNA-2/miRNA-155	BOS	Increased	—		Budding et al. [85]
MIP-1*α*/CCL-3	BOS	—	Increased	B	Verleden et al. [86]

A: described by several studies; B: few reports.

**Table 4:** Patient's perspectives.

*Diagnosis*
Where do I get the right diagnosis?
Where can I find help and who can I approach if I realize that my child remains unwell?
What does it do to my child and our family when the correct diagnosis is established?
Where can I get any specific information?
What is the long-term prognosis? Will my child die?
*Overall care*
What kind of therapy is available for my child and is there a cure?
Does my child need regular physiotherapy and is any specific physiotherapy required?
Does my child need psychological support and where would it be available?
Do we have to carry all the costs for medical and supportive therapy? Is there any support for travel costs?
*Family-related*
How often do we need to see a specialised center? How far do we have to drive to get to a center? Will I need to take off work for every appointment?
How can I talk to my child about the illness?
What impact does a chronic disease have to family life? Where can we go if we do not manage it?
What impact has the illness to the siblings? Will there be any support for them?
*Harm*
What side effects do the treatments have?
What implications does a chronic disease have for my child's future life?
Will my child be able to live on his own and will my child be able to live on own responsibility?
Does the diagnosis limit my child in his career choice?
Can my child undergo a normal teenage life with sport, leisure, party, stress?
